# MTNR1B Genetic Variability Is Associated with Gestational Diabetes in Czech Women

**DOI:** 10.1155/2014/508923

**Published:** 2014-07-15

**Authors:** Daniela Vejrazkova, Petra Lukasova, Marketa Vankova, Josef Vcelak, Olga Bradnova, Veronika Cirmanova, Katerina Andelova, Hana Krejci, Bela Bendlova

**Affiliations:** ^1^Department of Molecular Endocrinology, Institute of Endocrinology, 11694 Prague 1, Czech Republic; ^2^Institute for Mother and Child Care, Prague, 14710 Prague 4, Czech Republic; ^3^Department of Obstetrics and Gynecology, First Faculty of Medicine, Charles University and General University Hospital in Prague, 12000 Prague 2, Czech Republic

## Abstract

The gene *MTNR1B* encodes a receptor for melatonin. Melatonin receptors are expressed in human *β*-cells, which implies that genetic variants might affect glucose tolerance. Meta-analysis confirmed that the rs10830963 shows the most robust association. The aim of the study was to assess the rs10830963 in Czech GDM patients and controls and to study relations between the SNP and biochemical as well as anthropometric characteristics. Our cohort consisted of 880 women; 458 were diagnosed with GDM, and 422 were normoglycemic controls without history of GDM. Despite similar BMI, the GDM group showed higher WHR, waist circumference, abdominal circumference, and total body fat content. The risk allele G was more frequent in the GDM group (38.3 versus 29.4% in controls, OR 1.49 CI95% [1.22; 1.82]; *P*_OR_ = 0.0001). In spite of higher frequency, the G allele in the GDM group was not associated with any markers of glucose metabolism. In contrast, controls showed significant association of the allele G with FPG and with postchallenge glycemia during the oGTT. Frequency analysis indicates that rs10830963 is involved in gestational diabetes in Czech women. However, the association of the SNP with glucose metabolism, which is obvious in controls, is covert in women who have experienced GDM.

## 1. Introduction

The gene* MTNR1B* encodes a receptor for melatonin, the main regulator of the sleep cycle, circadian rhythm, and seasonal periodicity. Melatonin is secreted primarily by the pineal gland and is also released from the gastrointestinal tract [1]. Its levels rise over the night and fall gradually during the day [2]. Melatonin receptor 1B (MTNR1B) belongs to the G protein-coupled receptors, a large family consisting of more than 800 members in humans [3]. Melatonin receptors are expressed mainly in the brain, but* MTNR1B* has also been found in the human liver, kidney, adipose tissue, and pancreatic islets of Langerhans [2, 4], primarily in *β*-cells [5]. This finding implies that genetic variants in the* MTNR1B* might affect pancreatic glucose sensing, insulin secretion, and, conceivably, glucose tolerance. It is well documented that disturbances in circadian rhythm can result in impaired metabolism of glucose [6, 7]. Furthermore, a functional link between insulin and melatonin was reported [2]. Indeed, an association between the* MTNR1B* genetic variant and insulin secretion had been reported shortly afterwards [5]. Further associations between the melatonin receptor variants and increased fasting plasma glucose (FPG) and type 2 diabetes mellitus (T2DM) risk were described in genome-wide association study report (GWAS report) in 2009 [8, 9]. Follow-up studies and a meta-analysis confirmed that the intronic variant rs10830963 shows the most robust association with T2DM [10]. The effect of the allele G of this single nucleotide polymorphism (SNP) on FPG was replicated in children and adolescents, suggesting an early impact of the SNP during development [11, 12]. It was also reported in a longitudinal study that normal subjects carrying allele G of the rs10830963 are more likely to become glucose intolerant manifesting impaired fasting glucose, but the influence of the SNP on the clinical transition from impaired fasting glucose to overt T2DM was weaker [13]. Although the intronic location of the rs10830963 variant in an unconserved genomic region [14] does not provide any suggestion about its functional relevance, the genetic association of the SNP with FPG and T2DM is now very well documented, is replicated, and has reached a genome-wide significance across the Caucasian population [8, 9, 15, 16].

It is not surprising then that the association of rs10830963 with gestational diabetes mellitus (GDM) had been described [17, 18] and confirmed in some populations [19–22]. So far, the contribution to the effect of the SNP in Czech patients with GDM has not yet been published.

The aim of our study was to examine genetic, anthropometric, and biochemical differences between a group of young Czech women with history of GDM and a control group of Czech women with normal FPG levels without history of GDM. Our main intention in the genetic analysis was to assess and compare the* MTNR1B *gene SNP variant rs10830963 between both groups. We also aimed to study relations between this genetic locus and broad biochemical as well as anthropometric characteristics in both analyzed groups.

## 2. Materials and Methods

### 2.1. Study Subjects

Our cohort of studied subjects consisted of 880 adult women; 458 of them were diagnosed with GDM by the criteria based on WHO guidelines together with the Czech Diabetes Society and the Czech Gynecological and Obstetrical Society [23]. On the day of examination these women met the 0.5–2-year interval after childbirth. The control group was comprised of 422 women of similar age and BMI, without history of GDM and with normal fasting glucose levels <5.6 mmol/L. All the participants in the study were without other serious pathologies (i.e., hormonal disturbances, infections, organ disorders, mental illness, etc.). See more details concerning characteristics of studied subjects in Table 1. The study protocol was in accordance with institutional ethic guidelines and the national laws and all subjects gave their written informed consent to participate in the study.

### 2.2. *MTNR1B* Genotyping

DNA extracted from peripheral leukocytes (QIAamp DNA Blood Kit, QIAGEN, Germany) was used to genotype for rs10830963 variants by ABI TaqMan SNP Genotyping Assays (LightCycler 480 System, Roche).

### 2.3. Clinical and Biochemical Characterization

Body weight, height, and waist and hip circumferences were measured in all participants in order to calculate body mass index (BMI) and to evaluate body fat distribution by means of waist circumference and waist to hip ratio. Furthermore, body composition according to bioimpedance method (*Tanita AB-140 Viscan, Tanita BC-480)* was determined.

Venous blood samples were obtained after an overnight fast. Glucose metabolism was characterized by blood glucose (Beckman Glucose Analyser 2), immunoreactive insulin (Immunotech IRMA, Czech Rep.), C-peptide (Immunotech IRMA, Czech Rep.), proinsulin (DRG Diagnostics, Germany), and glucagon (IBL-International, Germany).

A 3-hour oral glucose tolerance test (oGTT) with 75 g of glucose load was performed in all subjects. Areas under the oGTT glycemic, C-peptide, and insulin curves (AUC) were calculated. Lipid profile was assessed by total cholesterol, high density lipoprotein, low density lipoprotein, and triglyceride concentrations (analyser Integra 400+, Roche Diagnostics GmbH, Germany). To assess insulin sensitivity, the following three indices were calculated: homeostasis model 1/HOMAR = 1/(insulin_0min⁡_ [*µ*U/mL] ×  glucose_0min⁡_ [mmol/L]/22.5), Matsuda index = 104/*√*(mean insulin_0min⁡_ [*µ*U/mL] × mean glucose_0min⁡_ [mmol/L] ×  glucose_0min⁡_ [mmol/L] ×  insulin_0min⁡_ [*µ*U/mL]), and Cederholm index = [75.000 + (glucose_0min⁡_ [mmol/L] −  glucose_120min⁡_ [mmol/L] × 1.15 × 180 × 0.19 × body weight [kg]]/[120 × log (mean insulin) × mean glucose [mmol/L]]. Beta-cell function was evaluated by HOMAF = 20 ×  insulin_0min⁡_ [*µ*U/mL]/(glucose_0min⁡_ [mmol/L] − 3.5).

Hormonal spectra (testosterone, dehydroepiandrosterone (DHEA), dehydroepiandrosterone sulfate (DHEAS), androstenedione, estradiol, prolactin, luteinizing hormone, follicles-stimulating hormone, and sex hormone binding globulin) were assessed due to* GC-MS, RIA, *or* ELISA *methods. Moreover, thyroid hormones TSH, fT3, and fT4 and liver enzymes ALS, AST, and GGT were evaluated (*Cobas 6000*).

In addition, questionnaires monitoring demographic and anamnestic data regarding family T2DM or gestational diabetes incidence were collected from all participants.

### 2.4. Statistical Analysis

To assess deviation from the Hardy-Weinberg equilibrium of the genotype frequencies, the Chi-square test [24] was used. Allele/genotype frequencies were compared between the two groups by Chi-square test. Odds ratios and 95% confidence intervals were calculated according to MedCalc software. Spearman correlation matrix was applied to assess relations between continuous variables. Differences in biochemical and anthropometric data between the compared groups were tested by nonparametric Mann-Whitney test owing to the nonnormal data distribution. The power analysis was conducted using the NCSS2004/PASS software. The* P* values <0.05 (two-tailed) were considered to be significant. Population-attributable risk (PAR) was calculated as PAR = (*X* − 1)/*X*, assuming the multiplicative model where *X* = (1 − *f*)^2^ + 2*f*(1 − *f*)*γ* + *f* ^2^ *γ* ^2^; *γ* is the odds ratio and* f* is the frequency of the risk allele G.

## 3. Results

### 3.1. Comparison of GDM and Control Group

#### 3.1.1. Anthropometry

The compared groupshad very similar BMI, but different body fat distribution: the GDM group showed significantly higher waist circumference, abdominal circumference, WHR, and also higher total body fat content measured by bioimpedance; see Table 1. Visceral fat rating according to the Viscan machine was higher in the GDM group (7.7  ±  9.31 in GDM group versus 5.7  ±  3.09 in control group; *P* = 0.01).

#### 3.1.2. Biochemistry

In biochemical parameters, there were differences in triglyceride concentration, liver enzyme AST, and testosterone between the two groups, although neither the GDM nor the control group exceeded the limit of the clinically normal range (Table 1). Regarding glucose metabolism, the GDM group had higher FPG but, in average, still within normal range, that is, <5.6 mmol/L (Table 1). Measurement of glucose levels during the oGTT revealed that the GDM group had significantly higher postchallenge glucose levels in 30, 60, 90, 120, and 150 min of the 3-hour testing. In brief, this observation is expressed as higher AUC_Glc7_ (area under the glycemic 7-point curve) values in the GDM group (Table 1). Basal fasting insulinemia did not differ between the GDM and control group; however, after the 75 g load of glucose the insulin levels were significantly higher in the GDM group from 30 to 120 min. This is reflected in higher AUC_Ins7_ (area under the insulinemic 7-point curve) in the GDM group (Table 1).

The different response of the two groups to the glucose load is apparent also in lower indices of insulin sensitivity (IS-Matsuda, IS-Cederholm) and in lower homeostasis model of *β*-cell function (HOMAF) index [25] in the GDM group (Table 1).

#### 3.1.3. Genetics

Distribution of genotypic frequencies of the* MTNR1B* SNP rs10830963 did not deviate from Hardy-Weinberg equilibrium (Chi-squared = 1.42; *P* = 0.48). Genotypic distribution as well as frequency of the risk conferring minor allele G was compared between the two groups of women. We found more heterozygous risk allele G carriers in the GDM group (49.6% versus 43.6% in controls) and also the GG homozygotes were more common in the GDM group (13.5% versus 7.6% in controls, Chi-squared = 16.28; test power 0.96; *P* = 0.0003 (Table 2)). Accordingly, the allele G was significantly more frequent in the GDM group (38.3% versus 29.4% in controls; Chi-squared = 15.55; test power 0.95; odds ratio 1.49 CI 95% [1.22; 1.82]; *P*_OR_ = 0.0001). The population-attributable risk for the allele G under the multiplicative model was in our study calculated to be 26%.

### 3.2. Association of the SNP rs10830963 with Biochemical and Anthropometric Data

#### 3.2.1. GDM Group

Despite higher frequency, the G allele in the GDM group was not associated with any of the basic markers of glucose metabolism: no differences between the particular CC, CG, and GG genotypes were observed in fasting glycemia, fasting insulinemia, C-peptide, indices of insulin sensitivity (Matsuda index, Cederholm index), and *β*-cell function (HOMAF index). There were even no significant differences in postchallenge levels of glycemia and insulinemia during the 3-hour oGTT testing between the genotypes in the GDM group.

Notably, neither FPG nor postchallenge glycemia levels correlated with the length of the postpartum period, that is, 0.5–2-year interval. The proportion of the variation in fasting glycemia can in our results be accounted for only as 0.3% by variation in the postpartum period (*r* = 0.05) and proportion of the variation in postchallenge glycemia concentration during the oGTT expressed as AUC_Glc7_ can be accounted for as 2.6% by variation in length of time after the childbirth (*r* = −0.16). A comparison of FPG between breastfeeding and nonbreastfeeding mothers in the whole GDM group showed a difference of borderline significance (4.8 ± 0.61 mmol/L in breastfeeding versus 4.9 ± 0.72 mmol/L in nonbreastfeeding; *P* = 0.03); no relationship with genotypes was observed.

Genotypes CC, CG, and GG in gestational diabetics did not differ in other biochemical data such as blood lipids, steroids, thyroid hormones, and, for thoroughness, any of the measured anthropometric parameters (BMI, WHR, waist and abdominal circumferences, and % of body fat); see Table 3.

#### 3.2.2. Control Group

**Figure 1:**
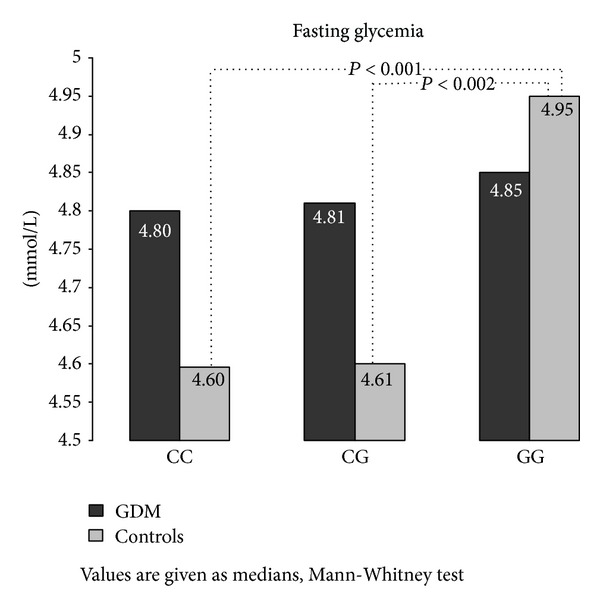
Fasting glycemia based on the* MTNR1B* SNP rs10830963 genotypes.

**Figure 2:**
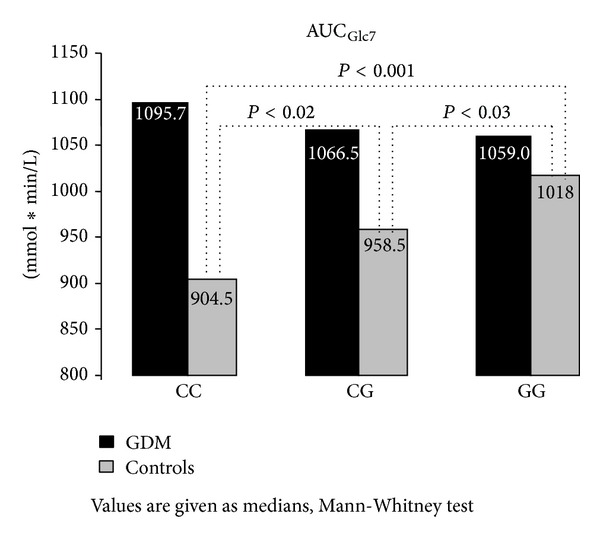
Area under the glycemic curve during the 7-point oGTT based on the* MTNR1B* SNP rs10830963 genotypes.

The observation in the group of controls was quite different. This group of women showed significant differences between the rs10830963 genotypes in fasting glycemia (Figure 1) as well as in postchallenge levels of glycemia throughout the oGTT; these differences were statistically significant in 30, 60, and 90 min of the test. This can be observed as different AUC_Glc7_ values between the genotypes in the controls (Figure 2). Fasting insulinemia was similar across the genotypes; however, postchallenge insulinemia levels in controls were higher in GG homozygotes compared with CC genotype in 90 min (48.5 ± 6.42 mIU/L versus 36.8 ± 25.06 mIU/L, resp.; *P* = 0.05) and 180 min (10.8 ± 9.11 mIU/L versus 6.9 ± 6.42 mIU/L, resp.; *P* = 0.04) of oGTT; these results corresponded well with the C-peptide concentrations (data not shown). Consequently, Cederholm index of insulin sensitivity was higher in CC homozygotes in comparison with risk GG genotype (80.3 ± 21.63 versus 69.2 ± 17.72, resp.; *P* = 0.02), as well as HOMAF index (184.8 ± 540.60 versus 122.6 ± 124.07, resp.; *P* = 0.05).

Statistically or clinically significant differences between the genotypes in the group of control women were not found in other biochemical parameters or in anthropometric data (Table 3).

## 4. Discussion

Numerous studies on ethnically diverse populations suggest that SNP rs10830963 is associated with a higher risk of GDM [10, 19–21]. A meta-analysis performed to estimate the association between the rs10830963 minor allele G and T2DM indicates a consistent and significant association in Caucasians, but not in Asians [26]. Limited cross-ethnicity is observed also as regards the influence of the allele on FPG. Several GWAS studies have verified that the rs10830963 allele G increases fasting glycemia levels in Europeans [8, 9, 15, 16, 27–29]. In Asian populations, the situation is less clear. While some studies have observed association of the rs10830963 allele G with increased FPG [30, 31], other studies focused on FPG testing made on Asians have not pointed to this SNP [32, 33]. In addition to ethnicity, a question also arises regarding the biochemical profile of the selected cohort, that is, normoglycemic subjects/nondiabetics/diabetics. Uncertainty persists especially with respect to functional implications of the intronic SNP rs10830963 in glucose homeostasis and diabetes development. The presented data resulting from identification and detailed study of quite rare cohort of risk GG homozygotes in GDM cases and controls demonstrate that a strong association of the SNP with GDM does not correspond with equivalently significant effect on FPG in the affected population, which concurrently contrasts with remarkable effect (though within physiological range) on FPG in healthy controls. Thus, in general, the allele associated with fasting glycemia in healthy individuals is not necessarily also associated with FPG in women with a history of GDM, or, conceivably, in subjects with impaired glucose tolerance or in type 2 diabetics, as GDM and T2DM share a similar genetic background [19]. Consistent results concerning G allele association with elevated FPG in nondiabetic individuals have been previously experienced [30, 34, 35].

How to interpret the results obtained in our study? GDM is characterized by a relatively diminished insulin secretion coupled with a pregnancy-induced insulin resistance. The final shift towards GDM during pregnancy is the result of the interplay between suboptimal changes in expression of numerous genes, epigenetic regulations, receptor signaling, many steroid hormones, cytokine production, and other regulatory factors. Without understanding functional relevance of the SNP, we can only speculate about the existence of some protective genetic or epigenetic factors present in risk-conferring GG genotype carriers from the control group preventing them from progressing to manifest GDM during pregnancy, which are not present in those who eventually developed the disease after they became pregnant. Concurrently, we can assume that there are other unrecognized epi/genetic prodiabetic cofactors present in gestational diabetics, which are missing in the controls. It is also highly probable that some consequences of complex changes resulting from pregnancy, delivery, lactation, and even a pathological condition such as GDM persist long after childbirth and may abolish tiny effect of the G allele on fasting and postchallenge glycemia, although we did not detect any correlation between glycemia and length of the postpartum period in our cohort of gestational diabetics. A several-year follow-up examination is necessary in order to understand to what extent the results are affected by quite short postpartum period ranging 0.5–2 years.

## 5. Conclusions

In conclusion, our study on a Czech cohort of women confirms that allele G of rs10830963 in* MTNR1B* gene is associated with increased risk of developing GDM and, in nondiabetic normoglycemic subjects, with FPG levels and glucose processing during the oral glucose-tolerance test. The explanation why the locus exerts apparent influence on healthy women, while it is undetectable in women with a history of GDM, remains speculative and will be examined further in the intended longitudinal study.

## Figures and Tables

**Table 1:** Subject characteristics.

Group	GDM	Controls	*P*
*n*	458	422	—
Age (years)	34.1 ± 6.12	34.8 ± 15.09	—

Anthropometry			
BMI (kg/m^2^)	24.3 ± 4.93	23.7 ± 4.18	0.28
Waist circ. (cm)	78.8 ± 10.80	75.1 ± 10.47	**<0.0001**
WHR	0.782 ± 0.05	0.747 ± 0.06	**<0.0001**
Abdominal circ. (cm)	87.1 ± 10.51	82.7 ± 10.98	**<0.0001**
Total body fat (%)	30.9 ± 7.99	27.8 ± 7.87	**0.008**

Biochemistry			
Basal glycemia (mmol/L)	5.03 ± 1.325	4.62 ± 0.403	**<0.0001**
AUC_Glc7_ (mmol∗min/L)	1097 ± 214	966 ± 177	**<0.0001**
Basal insulinemia (mIU/L)	7.37 ± 6.43	7.25 ± 5.04	0.26
AUC_Ins7_ (mIU∗min/L)	6740 ± 4972	5757 ± 3492	**0.0008**
HOMAF (mIU/mmol)	112.7 ± 86.31	141.7 ± 106.9	**<0.0001**
IS-Matsuda	8.0 ± 3.92	9.0 ± 4.19	**0.004**
IS-Cederholm	64.5 ± 18.03	77.4 ± 20.5	**<0.0001**
Cholesterol (mmol/L)	4.6 ± 0.89	4.6 ± 0.94	0.69
HDL (mmol/L)	1.6 ± 0.39	1.6 ± 0.37	0.95
LDL (mmol/L)	2.6 ± 0.86	2.6 ± 0.85	0.32
TG (mmol/L)	0.94 ± 0.686	1.01 ± 0.544	**<0.0001**
Testosterone (nmol/L)	1.61 ± 0.621	1.84 ± 0.765	**<0.0001**
DHEA (nmol/L)	17.9 ± 10.36	19.7 ± 12.48	0.16
DHEAS (umol/L)	4.3 ± 1.98	4.4 ± 2.51	0.99
Androstenedione (nmol/L)	6.4 ± 2.19	6.3 ± 2.75	0.16
ALT (ukat/L)	0.32 ± 0.159	0.31 ± 0.177	0.31
AST (ukat/L)	0.36 ± 0.139	0.38 ± 0.137	**0.01**
GGT (ukat/L)	0.27 ± 0.265	0.27 ± 0.201	0.21
TSH (mIU/L)	3.2 ± 4.75	2.5 ± 1.39	0.99
fT3 (pmol/L)	5.0 ± 0.83	4.9 ± 1.21	0.05
fT4 (pmol/L)	15.4 ± 3.07	15.5 ± 3.52	0.92

Values are given as mean ± SD, Mann-Whitney test.

**Table 2:** Genotypic and allelic frequencies of the *MTNR1B* SNP rs10830963.

Group/ genotype	CC	CG	GG	G allele freq.
GDM	169 (36.9%)	227 (49.6%)	62 (13.5%)	0.38
Controls	206 (48.8%)	184 (43.6%)	32 (7.6%)	0.29

Total	375 (42.6%)	411 (46.7%)	94 (10.7%)	0.34

Chi-squared for genotypic distribution = 16.28; *P* = 0.0003.

**Table 3:** Subject characteristics based on *MTNR1B* SNP rs10830963 genotypes.

Group	GDM			Controls		
Genotype	CC	CG	GG	CC	CG	GG
BMI (kg/m^2^)	24.9 ± 5.05	24.0 ± 4.77	23.8 ± 5.05	23.7 ± 4.16	23.7 ± 4.21	23.3 ± 4.20
Waist circ. (cm)	79.7 ± 10.51	78.1 ± 10.61	78.6 ± 12.31	75.3 ± 10.63	75.2 ± 10.25	74.6 ± 10.98
WHR	0.78 ± 0.06	0.78 ± 0.05	0.78 ± 0.05	0.75 ± 0.07	0.75 ± 0.06	0.75 ± 0.07
Abdominal circ. (cm)	88.3 ± 10.00	86.7 ± 10.79	85.4 ± 10.77	83.0 ± 11.08	82.6 ± 10.95	81.7 ± 10.80
Total body fat (%)	31.7 ± 7.90	30.4 ± 8.28	30.5 ± 7.43	27.4 ± 8.21	28.5 ± 7.59	26.1 ± 7.48

Cholesterol (mmol/L)	4.68 ± 0.821	4.67 ± 0.911	4.43 ± 1.025	4.65 ± 0.92	4.58 ± 0.95	4.68 ± 0.98
HDL (mmol/L)	1.57 ± 0.397	1.63 ± 0.415	1.61 ± 0.317	1.59 ± 0.375	1.60 ± 0.377	1.66 ± 0.331
LDL (mmol/L)	2.69 ± 0.794	2.63 ± 0.879	2.56 ± 0.981	2.65 ± 0.837	2.49 ± 0.840	2.59 ± 1.102
TG (mmol/L)	0.92 ± 0.435	0.92 ± 0.734	0.78 ± 0.419	1.02 ± 0.641	0.99 ± 0.407	1.07 ± 0.589

Testosterone (nmol/L)	1.55 ± 0.61	1.66 ± 0.64	1.59 ± 0.59	1.78 ± 0.753	1.87 ± 0.770	2.05 ± 0.794
DHEA (nmol/L)	16.8 ± 9.93	18.4 ± 10.63	19.7 ± 10.44	19.0 ± 11.24	20.7 ± 13.96	18.3 ± 10.96
DHEAS (umol/L)	4.5 ± 1.97	4.1 ± 1.98	4.31 ± 2.01	4.4 ± 2.50	4.6 ± 2.62	4.1 ± 1.84
Androstenedione (nmol/L)	6.3 ± 2.44	6.4 ± 2.07	6.6 ± 1.87	6.2 ± 2.67	6.4 ± 2.87	6.7 ± 2.57

ALT (ukat/L)	0.32 ± 0.141	0.32 ± 0.169	0.34 ± 0.176	0.30 ± 0.165	0.32 ± 0.177	0.34 ± 0.234
AST (ukat/L)	0.35 ± 0.114	0.37 ± 0.164	0.34 ± 0.09	0.38 ± 0.132	0.39 ± 0.142	0.38 ± 0.141
GGT (ukat/L)	0.30 ± 0.291	0.25 ± 0.269	0.23 ± 0.119	0.26 ± 0.184	0.27 ± 0.202	0.32 ± 0.281

TSH (mIU/L)	3.3 ± 5.69	3.0 ± 4.37	3.3 ± 2.96	2.7 ± 1.44	2.3 ± 1.15	2.9 ± 2.11
fT3 (pmol/L)	5.1 ± 0.76	5.0 ± 0.77	5.1 ± 1.17	4.9 ± 1.17	5.0 ± 1.28	5.2 ± 1.02
fT4 (pmol/L)	15.4 ± 3.85	15.3 ± 2.55	15.2 ± 2.33	15.6 ± 3.13	15.5 ± 4.05	15.4 ± 2.48

Values are given as mean ± SD.

## Abbreviations

AUC: Area under the curve
BMI: Body mass index
DHEA: Dehydroepiandrosterone
DHEAS: Dehydroepiandrosterone sulfate
FPG: Fasting plasma glucose
GWAS: Genome-wide association study
HOMA: Homeostasis models assessment
MTNR1B: Melatonin receptor 1B
oGTT: Oral glucose-tolerance test
PAR: Population-attributable risk
SNP: Single nucleotide polymorphism
GDM: Gestational diabetes mellitus
T2DM: Type 2 diabetes mellitus.
